# A19 INVESTIGATING MECHANISMS THAT DRIVE SYMPTOMS IN IRRITABLE BOWEL SYNDROME PATIENTS WITH PERCEIVED GLUTEN SENSITIVITY

**DOI:** 10.1093/jcag/gwac036.019

**Published:** 2023-03-07

**Authors:** C Seiler, G Rueda, P Miranda, A Nardelli, R Borojevic, D Schuppan, P Moayyedi, E Verdu, S Collins, M I Pinto-Sanchez, P Bercik

**Affiliations:** 1 Farncombe Institute, McMaster University, Hamilton, Canada; 2 Johannes-Gutenberg-University, Mainz, Germany; 3 Beth Israel Deaconess Medical Center, Boston, United States

## Abstract

**Background:**

Patients with irritable bowel syndrome (IBS) often report gastrointestinal symptoms after consuming wheat and gluten-containing foods. It is, however, unclear whether gluten is the main driver of symptoms, as other immunogenic peptides, such as amylase trypsin inhibitors (ATI), poorly digestible fiber (inulin, part of FODMAP) or even the nocebo effect may contribute to symptom generation.

**Purpose:**

To evaluate whether whole wheat containing ATIs and/or purified gluten trigger gastrointestinal symptoms compared to nocebo in patients with IBS adopting a gluten-free diet (GFD).

**Method:**

We conducted a double-blind, randomized, nocebo-controlled crossover study in adult IBS patients (Rome IV criteria) who previously perceived improvement of symptoms while on a GFD. The study was approved by the Hamilton Research Ethics Board (HiREB #4367). Participants were challenged for 7 days with whole wheat, purified gluten, and nocebo (gluten-free flour) added to low FODMAP cereal bars. Each challenge was followed by a 2-week washout. Patients remained on a GFD throughout the study, diet adherence was assessed by a dietitian and stool gluten immunogenic peptides (GIP; Biomedal). Gastrointestinal symptoms were assessed by IBS Symptom Severity Score (IBS-SSS); increases >50 points were considered a significant worsening. Blood samples were collected to assess immune markers and celiac (HLA DQ2, DQ8 and DQ7) genotype. Statistical comparisons used Friedman rank sum tests and paired Wilcoxon signed rank tests.

**Result(s):**

Twenty-nine IBS patients (27 female, mean age=42, SD=14.4 years) were enrolled in the study; 1 dropped. Similar proportions of patients reacted symptomatically to wheat (11/28, 39.3%), gluten (10/28, 35.7%) and nocebo (8/28, 28.6%). However, there was an overall significant increase in IBS symptoms after wheat (+39.5 on IBS-SSS; p=0.030) but not after gluten (+27.5; p=0.051) or nocebo (+5.5; p=0.236) challenges (Figure 1). Ten participants experienced IBS-SSS symptoms >175 during baseline and did not worsen further during the challenges. TNF-α trended from 1.35 pg/mL after nocebo, 1.47 pg/mL after gluten, to 1.57 pg/mL after wheat; however, this was not significant. Baseline adherence to a GFD was rated excellent in 19 (68%), good in 6 (21%), and fair in 3 (11%) participants. Median GIP levels were 0.584 µg/g after wheat, 0.432 µg/g after gluten, and 0.095 µg/g after nocebo; p<0.0001. Celiac predisposition genes were present in 19/24 participants (10/24 had DQ2, 2/24 had DQ8, and 9/24 had DQ7).

**Image:**

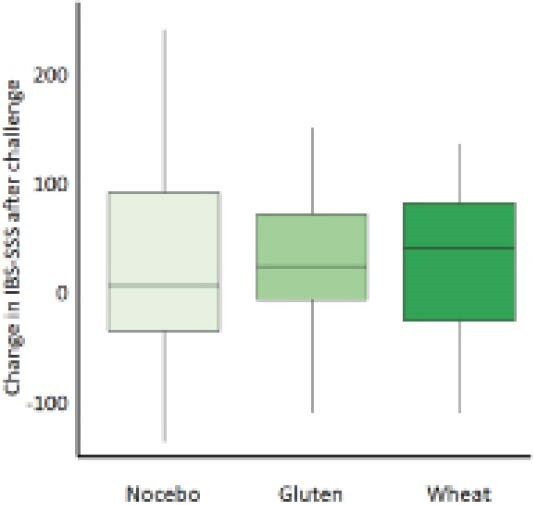

**Conclusion(s):**

IBS patients self-reporting wheat or gluten sensitivity had worse symptoms after whole wheat, but not purified gluten or nocebo challenge. However, similar proportions of IBS patients reacted to each intervention, suggesting that central mechanisms play an important role in symptom genesis. Furthermore, one third of patients had high symptoms during a GFD and did not react to wheat or gluten challenges, suggesting that other mechanisms are driving their IBS symptoms.

**Please acknowledge all funding agencies by checking the applicable boxes below:**

CIHR, Other

**Please indicate your source of funding;:**

Society for the Study of Celiac Disease (Nestle); Canadian Digestive Health Foundation

**Disclosure of Interest:**

None Declared

